# Ether-Based High-Voltage Lithium Metal Batteries: The Road to Commercialization

**DOI:** 10.1021/acsnano.4c00110

**Published:** 2024-04-11

**Authors:** Jingwei Xiang, Yi-Chun Lu

**Affiliations:** Electrochemical Energy and Interfaces Laboratory, Department of Mechanical and Automation Engineering, The Chinese University of Hong Kong, Shatin, Hong Kong SAR 999077, People’s Republic of China

**Keywords:** ether-based electrolyte, high-voltage, lithium metal batteries, lithium salts optimization, solvent engineering, functional additives, practical performance assessment, practical safety assessment

## Abstract

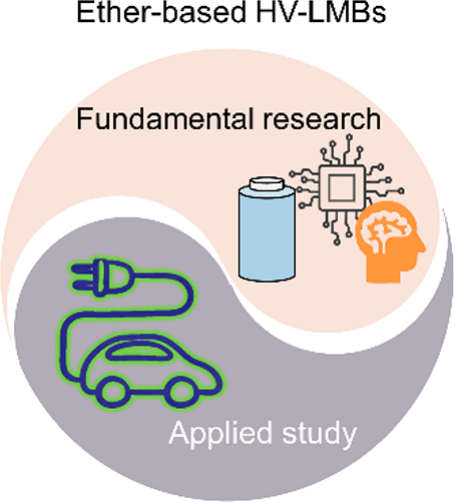

Ether-based high-voltage lithium metal batteries (HV-LMBs) are drawing growing interest due to their high compatibility with the Li metal anode. However, the commercialization of ether-based HV-LMBs still faces many challenges, including short cycle life, limited safety, and complex failure mechanisms. In this Review, we discuss recent progress achieved in ether-based electrolytes for HV-LMBs and propose a systematic design principle for the electrolyte based on three important parameters: electrochemical performance, safety, and industrial scalability. Finally, we summarize the challenges for the commercial application of ether-based HV-LMBs and suggest a roadmap for future development.

Commercial lithium-ion batteries (LIBs) are experiencing exponential growth in various emerging fields such as the electric vehicle and large-scale energy storage.^1−4^ Take electric vehicles as an example: the global demand for power LIBs was only 19 GWh in 2015, while by 2020, the demand had jumped to 170 GWh.^5,6^ However, in contrast to the fast-increasing demand for LIBs, the energy density of LIBs is growing very slowly. After decades of development, the energy density of LIBs is still far from the target of 500 Wh kg^–1^.^7−9^ Consequently, there is a pressing need to design next-generation battery systems with high energy densities.

Among various advanced battery systems, high-voltage lithium metal batteries (HV-LMBs ≥ 4.3 V vs Li/Li^+^) are expected to realize a breakthrough in energy density, achieving the 500 Wh kg^–1^ target.^10−12^ Nevertheless, the commercialization of HV-LMBs still faces many challenges, including the Li dendrite growth, deterioration of cathodes, and the decomposition of electrolyte, which can lead to poor cycle life and compromised battery safety. A particular safety concern is the growth of the Li dendrite leading to short circuits and thermal runaway (TR), causing severe safety risks.^13−15^ Currently, the electrolytes applied in LMBs can be divided into carbonate electrolytes,^16^ sulfone electrolytes,^17,18^ sulfonamide electrolytes,^19,20^ nitrile electrolytes,^21,22^ ionic liquid-based electrolytes,^23,24^ and ether electrolytes.^25,26^ Although carbonate electrolytes are widely used in conventional LIBs, they are difficult with which to form a stable solid–electrolyte interphase (SEI) on the surface of the Li metal anode, leading to the continuous growth of Li dendrites. While sulfone electrolytes have good oxidation stability and high dielectric constants, their high viscosity and poor stability to Li metal anode limit their application in LMBs. Sulfonamide electrolytes have been proven to exhibit improved performance in HV-LMBs compared to carbonate electrolytes. However, the weak coordination ability of this type of electrolytes with lithium ions results in low ion conductivity, thereby sacrificing the rate performance of LMBs. Nitrile electrolytes exhibit poor reductive stability with Li metal anode and cannot meet the demand of long-life LMBs. Ionic liquid-based electrolytes are also difficult to be widely used in LMBs due to their high price and high viscosity. Compared to the various electrolytes mentioned above, ether electrolytes are considered highly suitable for application in LMBs due to their excellent compatibility with the Li metal anode, high conductivity, low viscosity, and low cost. Unfortunately, the oxidative stability of ether-based electrolytes in standard lithium salt concentrations (∼1 M) is very low (<4.0 V vs Li/Li^+^).^25−27^ Therefore, it is crucial to enhance the oxidative stability of the electrolyte and build a robust cathode–electrolyte interphase (CEI) on the cathode to enable the stable application of ether-based HV-LMBs.

Recently, great efforts have been made to enhance the stability of electrolytes in ether-based HV-LMBs. The relevant strategies can be classified into three types (Figure 1a). The first one is to regulate the concentration and the type of lithium salts to reduce the proportion of free ether solvents, thereby constructing a stable CEI primarily formed by the decomposition of lithium salts.^28−32^ The second one is to improve the oxidative stability of the electrolyte by altering the structure of solvent molecules such as the introduction of fluorinated groups.^33−35^ The third one is to inhibit the decomposition of ether solvents at the interface by introducing functional additives.^36,37^ However, in addition to enhancing the oxidative stability of ether-based electrolytes themselves, the following three other aspects need to be further considered for the successful commercial application of ether-based HV-LMBs (Figure 1b). (1) Costs: the cost of LIBs will drop to $80 per kWh by 2030 based on the U.S. Department of Energy’s prediction.^38^ (2) Battery safety: researchers need to ensure that the battery suffers no TR under various abuse conditions.^39^ (3) Practical energy density: this should be considered due to limiting the amount of electrolyte usage and appropriate negative to positive capacity ratio (N/P ratio).^40^ Here, we discuss opportunities and future challenges of ether-based HV-LMBs in terms of both fundamental research and commercial application. In addition to focusing on the oxidative stability of electrolytes, more attention should be paid to evaluate whether the adopted strategies would lead to an increase in battery costs and whether the power density, energy density, cycle life, and safety of the HV-LMBs can meet the demands of commercial applications under harsh practical conditions. Based on this consideration, we propose a systematic design principle for ether-based electrolytes for HV-LMBs based on three important parameters: electrochemical performance, safety, and industrial scalability. Additionally, we discuss the development roadmap to guide the transition from ideas to commercial products.

**Figure 1:**
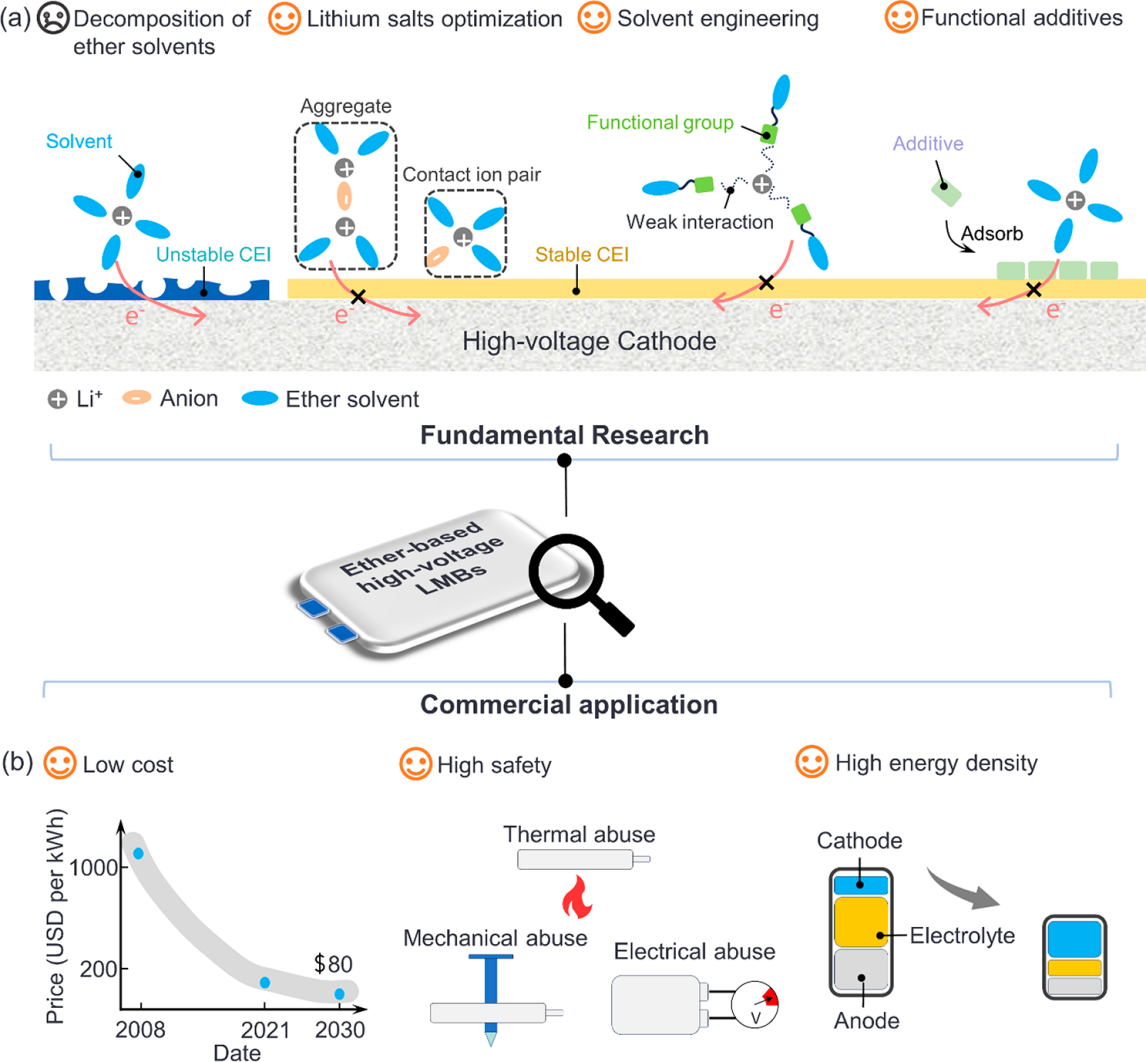
Schematic diagram of the (a) fundamental research and (b) commercial application for ether-based HV-LMBs.

## Fundamental Research

### Lithium Salts Optimization

**Figure 2:**
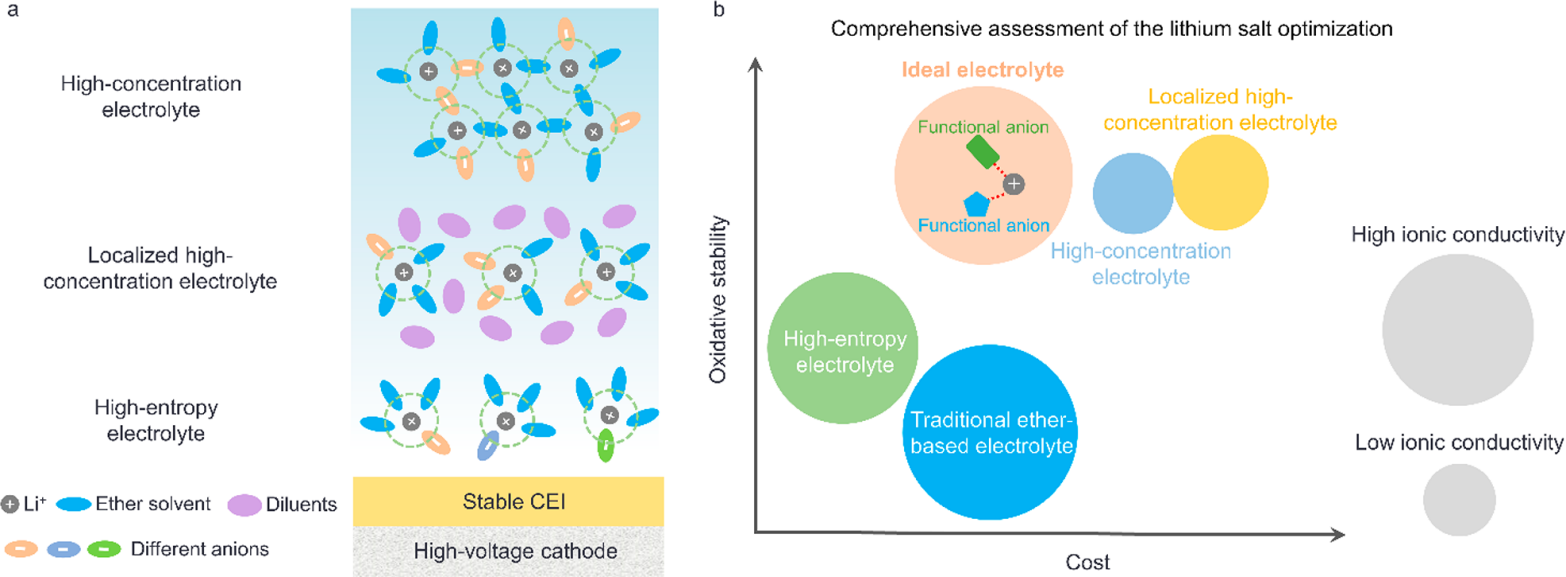
(a) Schematic of the solvation structures of ether-based HCEs, LHCEs, and high-entropy electrolytes for HV-LMBs. (b) Comprehensive assessment for lithium salt optimization.

As an important element of the electrolyte, the concentration and type of lithium salts have significant impacts on the viscosity, solvation structure of Li ions, and ionic conductivities. Simultaneously, the decomposition products of lithium salts during cycling are important components of the SEI and CEI, which contribute to the formation of dense and stable SEI and CEI.^41−43^ However, as the most widely used lithium salts, lithium hexafluorophosphate (LiPF_6_) is not suitable for ether-based HV-LMBs due to due to its difficulty in forming a stable SEI on the Li metal anode and high-voltage instability.^44,45^ The electrochemical performance of LMBs can be significantly improved by replacing LiPF_6_ with lithium bis(trifluoromethane sulfonyl) imide (LiTFSI) or lithium bis(fluorosulfonyl)imide (LiFSI) in ether-based electrolytes because the optimization of lithium salts can regulate the solvation structure of Li ions and improve the stability of SEI and CEI.^46^ Currently, lithium salt optimization strategies can be divided into two categories (Figure 2a): one is the lithium salt concentration adjustment, such as high-concentration electrolytes (HCEs)^47,48^ and the localized high-concentration electrolytes (LHCEs);^30,49,50^ the other is the regulation of lithium salt types, such as high-entropy electrolytes.^32,51^

Although HCEs have excellent oxidative stability, high lithium salt concentrations bring critical challenges for the commercial application of HCEs, such as the increased electrolyte viscosity, the low wettability of the electrolyte on the separator and electrode, and so on. Aiming to address these challenges of the HCEs, researchers design LHCEs by adding inert cosolvents as diluents to the HCEs.^30,52−54^ These diluents exhibit a wide electrochemical stability window, low viscosity, and extremely low solubility for lithium salts while remaining miscible with ether solvents. As a result, these diluents can significantly lower the lithium salt concentration of LHCEs while retaining contact ion pairs (CIPs) and aggregates (AGGs), ensuring that the electrolyte possesses a sufficiently high oxidative stability. Some highly fluorinated cosolvents, such as tris(2,2,2-trifluoroethyl) orthoformate (TFEO),^55,56^ 1,1,2,2-tetrafluoroethyl-2,2,3,3-tetrafluoropropylether (TTE),^49,57^ and 2H,3H-decafluoropentane (HFC),^58^ have been demonstrated to improve the stability of LHCE in HV-LMBs as diluents. Unfortunately, the addition of large amounts of expensive diluent to the electrolyte results in increased electrolyte cost as well as reduced ionic conductivity. In addition to commonly used hydrofluoroether diluents, some groups also reported low-cost fluorinated aromatic compounds as diluents.^59−61^ However, the low polarity of fluorinated aromatic compounds still limits their practical application as the addition of large quantities of these solvents could reduce the ionic conductivity of electrolytes.

Aside from the concentration of the lithium salt, the category of lithium salts is also a key factor in the performance of electrolytes. The application of mixtures of multiple lithium salts has been proven to enhance the oxidative stability of ether-based electrolytes.^32,62,63^ Compared to single-lithium salt electrolytes, the solvation structure with multiple lithium salts (high-entropy electrolyte) shows a high degree of disorder, resulting in weak interactions between lithium ions and ether solvents.^51^ Consequently, it contributes to the formation of an anion-derived CEI similar to that formed in HCEs. Compared with HCEs and LHCEs, high-entropy ether-based electrolytes have a lower cost and higher ionic conductivity.

The optimization of lithium salts has been demonstrated as a beneficial approach to improve the oxidative stability of ether-based electrolytes in HV-LMBs. In addition to oxidative stability, the cost and ion conductivity of electrolytes are also key factors in determining their commercial potential. For both HCE and LHCE, their high costs and low ionic conductivity constrain their commercial applications. As for the high-entropy electrolyte, it improves the oxidative stability without high-concentration lithium salts and the addition of highly fluorinated diluents. Nevertheless, the oxidative stability of high-entropy electrolytes needs to be further improved to satisfy the needs of long-cycling HV-LMBs. In the future, the lithium salt optimization strategy should comprehensively consider key factors such as the oxidative stability, cost, and ionic conductivity (Figure 2b). The rich experience in lithium salt design accumulated in other electrolyte systems, such as the 1,1,1-trifluoro-*N*-[2-[2-(2-methoxyethoxy)ethoxy)]ethyl] methanesulfonamide (LiFEA) with self-cleaning function,^64^ the 1, 1, 2, 2, 3, 3-hexafluoropropane-1, 3-disulfonimide (LiHFDF) capable of forming highly fluorinated SEI^65^ and CEI, and the lithium bis(*N*-nonafluorobutanesulfonyl)imide (LiNFSI) with a Teflon-like anion,^66^ can be utilized to design lithium salts with functional anions for ether-based HV-LMBs, achieving the enhancement of electrochemical performance under standard lithium salt concentration through the strategic combination of lithium salts with different functional anions.

### Solvent Engineering

**Figure 3:**
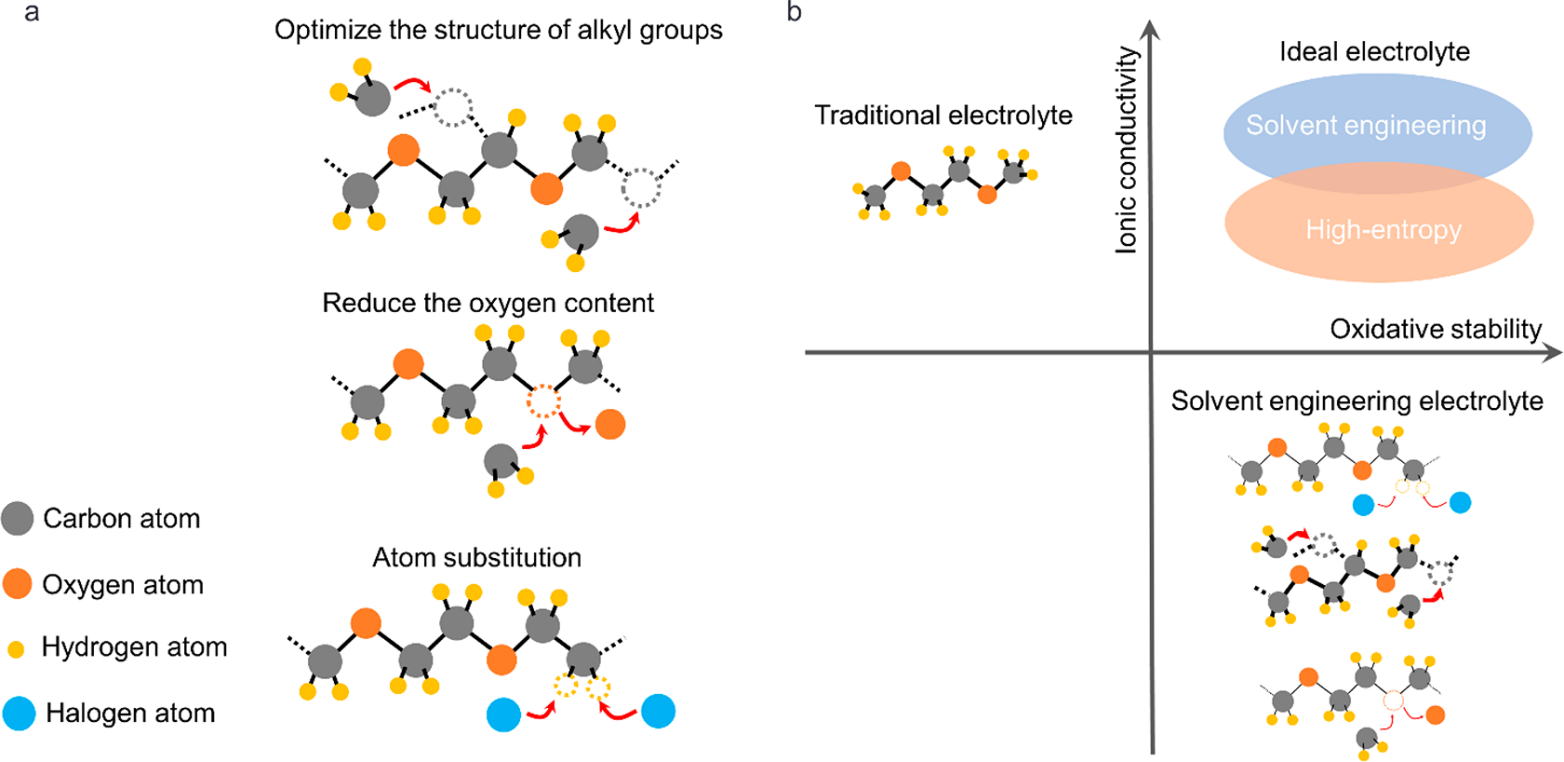
(a) Illustration of three different solvent engineering strategies for ether-based HV-LMBs. (b) Comparison of the ionic conductivity and oxidative stability of different electrolytes.

Compared to lithium salt optimization, solvent engineering is a more direct strategy to enhance intrinsic oxidative stability of ether solvents. At present, solvent engineering strategies can be categorized into three types (Figure 3a): the first one involves optimizing the structure of alkyl groups in solvent molecules,^67−72^ the second one is to reduce the oxygen content in solvent molecules,^73−75^ and the third is to perform atom substitution of the solvent molecules.^33,35,76−81^

The commonly used ether solvents, such as 1,2-dimethoxyethane (DME), exhibit poor oxidative stability and are not suitable for use in HV-LMBs. By increasing the alkyl length or introducing branched alkyl chains of DME molecules, the interaction between solvent and lithium ions can be weakened to promote the formation of CIPs and AGGs, which contributes to the development of a stable anion-derived CEI.^67,68^ The application of this strategy to other ether solvents has also been proven effective.^69,70^ Additionally, the solvation structure can be regulated via tuning the oxygen content of solvent molecules. It has been demonstrated by theoretical calculations and experiments that ether solvents with low oxygen contents exhibit weak coordination ability with lithium ions.^74,75^ The weak coordination facilitates the formation of anion-derived SEI and CEI, similar to those formed in HCEs and LHCEs, thus contributing to the enhancement of the interfacial stability. The strategy of replacing atoms in ether molecules with halogen atoms or halogen groups has proved as a highly promising strategy to improve the stability of ether-based electrolytes.^33,35,76−78^ Benefiting from the electron-withdrawing effect of halogen groups, their introduction can effectively inhibit the decomposition of ether solvents at a high voltage. Simultaneously, the incorporation of halogen atoms or halogen groups can change the lithium-ion solvation structure, thus facilitating anion-derived CEI and SEI formation.

In contrast to HCEs and LHCEs, solvent engineering can realize improvement in the oxidative stability of electrolytes without high-concentration lithium salts or the addition of highly fluorinated diluents. However, as shown in Figure 3b, whether it is optimizing the structure of alkyl groups, reducing the oxygen content, or introducing halogen atoms or groups, they all result in the decreased coordination ability of solvents with lithium ions, thereby reducing the ionic conductivity of electrolytes. Although the strategy proposed by Cui’s group, replacing fully fluorinated groups with partially fluorinated groups, can enhance the ionic conductivity,^35^ it still remains lower than that of traditional commercial electrolytes. Therefore, solvent engineering can be considered as sacrificing the ionic conductivity to enhance its oxidative stability. Fortunately, the strategy of preparing high-entropy electrolytes by mixing various ether solvents can significantly enhance the ionic conductivity of electrolytes, providing a promising strategy to addressing the aforementioned issue.^82^ However, it still employs expensive highly fluorinated ether solvents as cosolvents, which increases the cost of the electrolyte. Therefore, combining atom substitution with a high-entropy electrolyte is a promising research direction to achieve ideal ether-based electrolytes with high ionic conductivity, high oxidative stability, and low cost.

### Functional Additives

Compared to the two previously mentioned strategies, functional additives, such as LiNO_3_,^36,37,83,84^ triallyl phosphate (TAP),^85^ vinylene carbonate (VC),^86^ lithium difluoro(oxalate)borate (LiDFOB),^87^ lithiumtrifluoromethyl acetate (LiCO_2_CF_3_),^28^ etc., can improve the electrochemical performance of the electrolyte by regulating the components of SEI/CEI or the electrical double layer (EDL) without compromising cost and ionic conductivity. Among them, LiNO_3_ is currently one of the most reported additives. As an electrolyte additive, LiNO_3_ can help to build an N- and F-rich CEI and SEI to improve the stability of the interface. At the same time, NO_3_^–^ can enter the EDL, inhibiting the decomposition of ether solvents near the high-voltage cathode.^36,37^

Although there has been some progress in the research on functional additives, future development in this field still faces challenges. For example, LiNO_3_ has been proven to be a high-performance additive that simultaneously improve the stability of CEI and SEI in HV-LMBs. However, its ability to improve the oxidative stability of electrolytes is limited, and it is currently not able to satisfy the requirements of long cycling HV-LMBs with higher upper cutoff voltages (≥4.5 V vs Li/Li^+^). Although NO_3_^–^ has been demonstrated to enter the EDL at the beginning of cycles, continuous breakdown and repair of the CEI during cycling lead to the consumption and redistribution of NO_3_^–^ in the EDL, resulting in EDL instability during long cycling. Therefore, it is essential to choose suitable functional additives to realize long-term dynamic stability of EDL.

**Figure 4:**
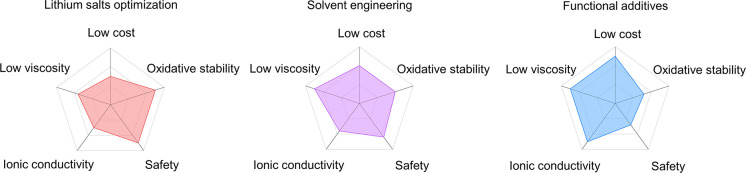
Comparison of key properties for three different ether-based electrolyte optimization strategies for HV-LMBs.

In summary, in future fundamental research on ether-based electrolytes for HV-LMBs, a comprehensive assessment of the designed electrolyte should be conducted based on the following five parameters (Figure 4): oxidative stability, ionic conductivity, cost, flammability, and viscosity. The strategies related to lithium salt optimization, such as HCEs and LHCEs, have been demonstrated to significantly enhance the oxidative stability of electrolytes. However, these electrolytes still face the challenges of low ionic conductivity and high cost. Recently, solvent engineering has received increasing attention because of its ability to enhance the oxidative stability of electrolytes under a standard lithium salt concentration. Nevertheless, these optimized solvent molecules often exhibit weak coordination with lithium ions, causing a reduced ionic conductivity of the electrolyte. Functional additives present a promising strategy for commercial application, as they can improve the oxidative stability of electrolytes without decreasing the ionic conductivity or increasing the cost of the electrolyte. However, the designed electrolytes with functional additives are still unable to satisfy the demands of long-life HV-LMBs. In the future, it can be considered to integrate the design of lithium salts, optimization of solvent molecules, and functional additives to achieve a comprehensive improvement in electrolyte performance.

## Commercial Application

Commercial applications require consideration of factors beyond the electrochemical performance of the battery, such as the scalability, battery safety, and cost.

### Industrial Scalability

With the continuous development of relevant technologies, the cost of commercial LIBs has been consistently decreasing in recent years. Compared to traditional LIBs, HV-LMBs experience a significant increase in cost due to the application of expensive Li metal anode (>$250 per kg) instead of the low-cost graphite (<$10 per kg).^88,89^ Therefore, it is necessary to strictly control the ratio of negative to positive capacity in a low range (<3), which can ensure that the HV-LMB has a high energy density and a low cost.^90^ The current price of carbonate-based electrolyte for commercial LIBs is less than $20 per kg, accounting for 5–10% of the battery cost.^91−93^ Although the designed HCEs and LHCEs are of important significance in gaining insight into the failure mechanisms of ether-based electrolytes for HV-LMBs, the use of high-concentration lithium salts and highly fluorinated diluents also increases the cost of the electrolyte. Therefore, to further promote the ether-based electrolyte for HV-LMBs from lab to market, consideration should be given to designing electrolytes that meet the demands of HV-LMBs under the conditions of a standard lithium salt concentration and with less highly fluorinated diluents.

Apart from the cost of the electrolyte, the scalability of the designed electrolyte preparation process also plays a crucial role in its commercialization.^7,89^ So far, researchers have employed solvent engineering to design various ether solvents with specific molecular structures. These designed ether solvents exhibit a good electrochemical performance in HV-LMBs without the addition of highly fluorinated diluents. However, due to the complicated preparation process of these designed solvents and low yields, the goal of large-scale preparation has not yet been achieved. Consequently, the design and preparation process of these designed ether solvents should be further optimized to improve the production efficiency.

With the continuous growth of the electrochemical energy storage market, future demand for electrolytes is also expected to experience exponential growth. Therefore, it is necessary to consider the environmental impact of the production and use of large quantities of electrolytes. Although organic halides, when applied as cosolvents or additives in electrolytes, help to improve the electrochemical performance of HV-LMBs, these organic halides are relatively stable in the ecosystem and are difficult to degrade naturally. Consequently, they will accumulate in the food chain and eventually do harm to humans, animals, and plants.^94,95^ Thus, the application of organic halides in ether-based electrolytes should be minimized to promote sustainable development of this field.

### Safety Assessment

In recent years, safety incidents of commercial LIBs have occurred frequently. Therefore, improving the safety of the battery has become a critical challenge in this field. Currently, safety incidents of LIBs are mainly induced by the TR of batteries under various abuse conditions.^39,96−98^ Compared to traditional LIBs, ether-based HV-LMBs exhibit an increased risk of TR for the following three reasons.^26,99,100^ (1) In contrast to graphite anode, Li metal anode is much more reactive with electrolytes to produce various reductive byproducts. These byproducts migrate to the oxidative cathode and undergo severe side reactions, which generate large amounts of heat and gas, thereby increasing the risk of TR. Simultaneously, the continued growth of Li dendrites during cycling increases the risk of internal short circuit. (2) As the upper cutoff voltage increases, the cathode will release more reactive oxygen and transition metal ions. When these substances migrate to the anode, they will destroy the SEI and react with the anode, producing massive amounts of gas and heat. (3) Compared to carbonate electrolytes, ether electrolytes exhibit poorer thermal stability, which makes it easier for the solvents to decompose and vaporize during the early stage of battery self-heating, thus exacerbating the heat accumulation inside batteries. Therefore, significant challenges still remain in improving the safety of ether-based HV-LMBs.

At present, there is limited research on the mechanism of TR and the safety assessment of ether-based HV-LMBs under practical conditions. Hence, it is essential to perform in-depth studies of the mechanisms of TR and establish comprehensive battery safety assessment standards. To date, some groups have achieved ether-based electrolytes nonflammable for HV-LMBs by adding flame retardant additives or introducing halogen elements into ether molecules.^101−103^ However, it has been demonstrated that the thermal runaway of batteries is not only related to the properties of the electrolyte itself but also closely linked to side reactions between the electrolyte and electrodes as well as the crosstalk of substances inside batteries. Even the battery using nonflammable electrolytes still experiences TR.^104,105^ Therefore, the following parameters need to be comprehensively evaluated in the safety assessment of ether-based HV-LMBs. (1) Safety concerns under thermal abuse conditions: conduct combustion experiments to test the flammability of electrolytes and use differential scanning calorimetry (DSC) to study the thermal stability of the electrolyte and the mixture of the electrolyte and electrodes. Additionally, accelerating rate calorimetry (ARC) is utilized to investigate the TR of the battery at different states of charge. (2) Safety concerns under electrical abuse conditions: investigate whether the battery is safe under overcharge and overdischarge conditions. (3) Safety concerns under mechanical conditions: perform nail penetration tests and drop tests were performed to verify the safety of the battery.

**Figure 5:**
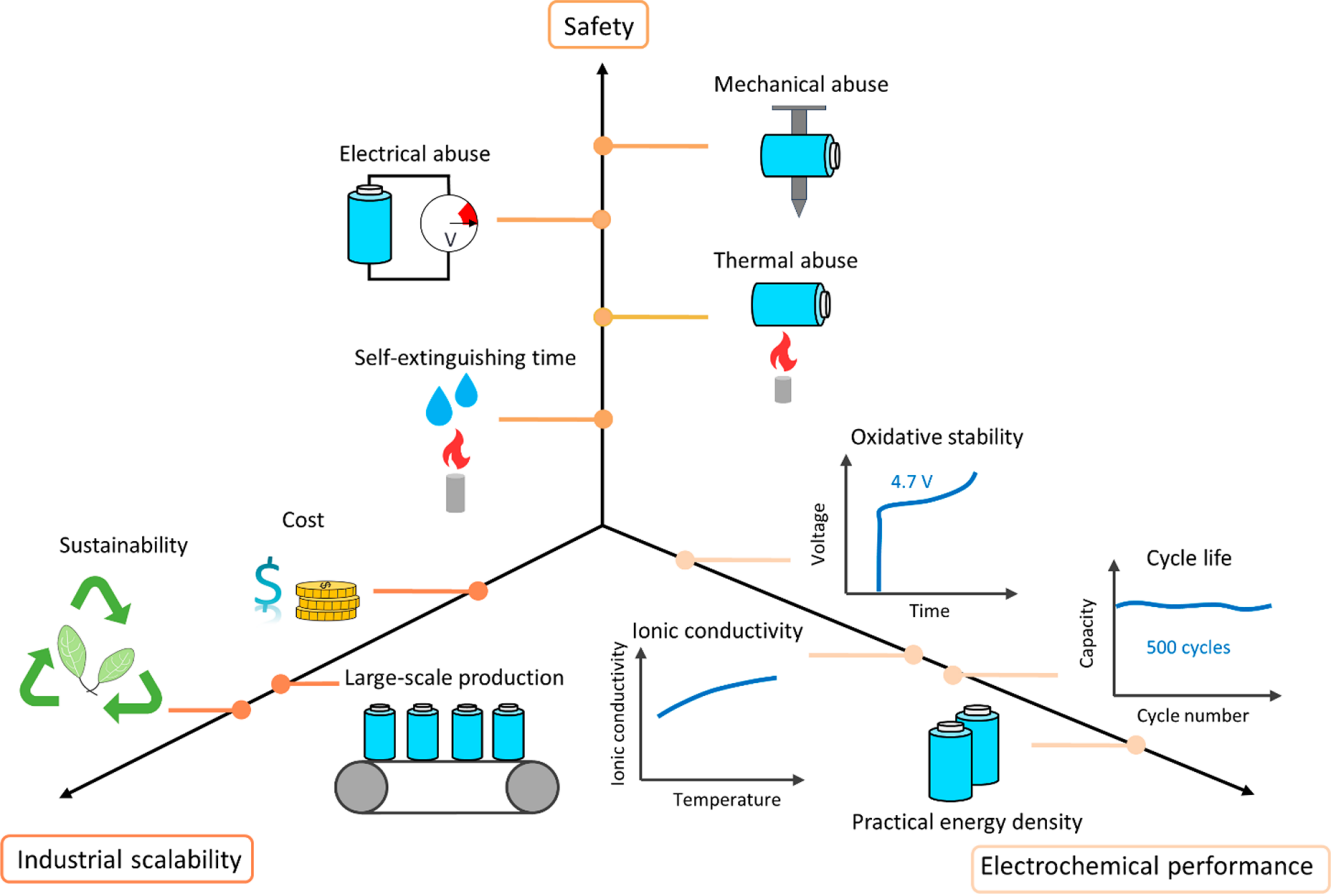
Proposed design principle for ether-based electrolytes for commercial HV-LMBs is based on electrochemical performance, safety, and industrial scalability.

Overall, apart from the electrochemical performance, factors such as the cost and safety of the ether-based HV-LMBs are also crucial for the future commercialization of the batteries. Based on this consideration, we propose a design principle for ether-based electrolytes applied in HV-LMBs. As shown in Figure 5, the designed ether-based electrolyte for HV-LMBs needs to be comprehensively evaluated from three dimensions. (1) Electrochemical performance: this part includes parameters such as the ionic conductivity and oxidative stability of the electrolyte, cycle life, rate performance, and energy density of batteries under practical conditions. (2) Safety: it contains the flammability of the electrolyte as well as the safety of the battery under various abuse conditions. (3) Industrial scalability: this covers the cost of battery raw materials, the scalability of battery production, the environmental impact assessment, and the sustainable development of this battery system. Following the proposed systematic design principle, the designed ether-based HV-LMBs will be closer to the requirements of commercial applications.

## Summary and Outlook

**Figure 6:**
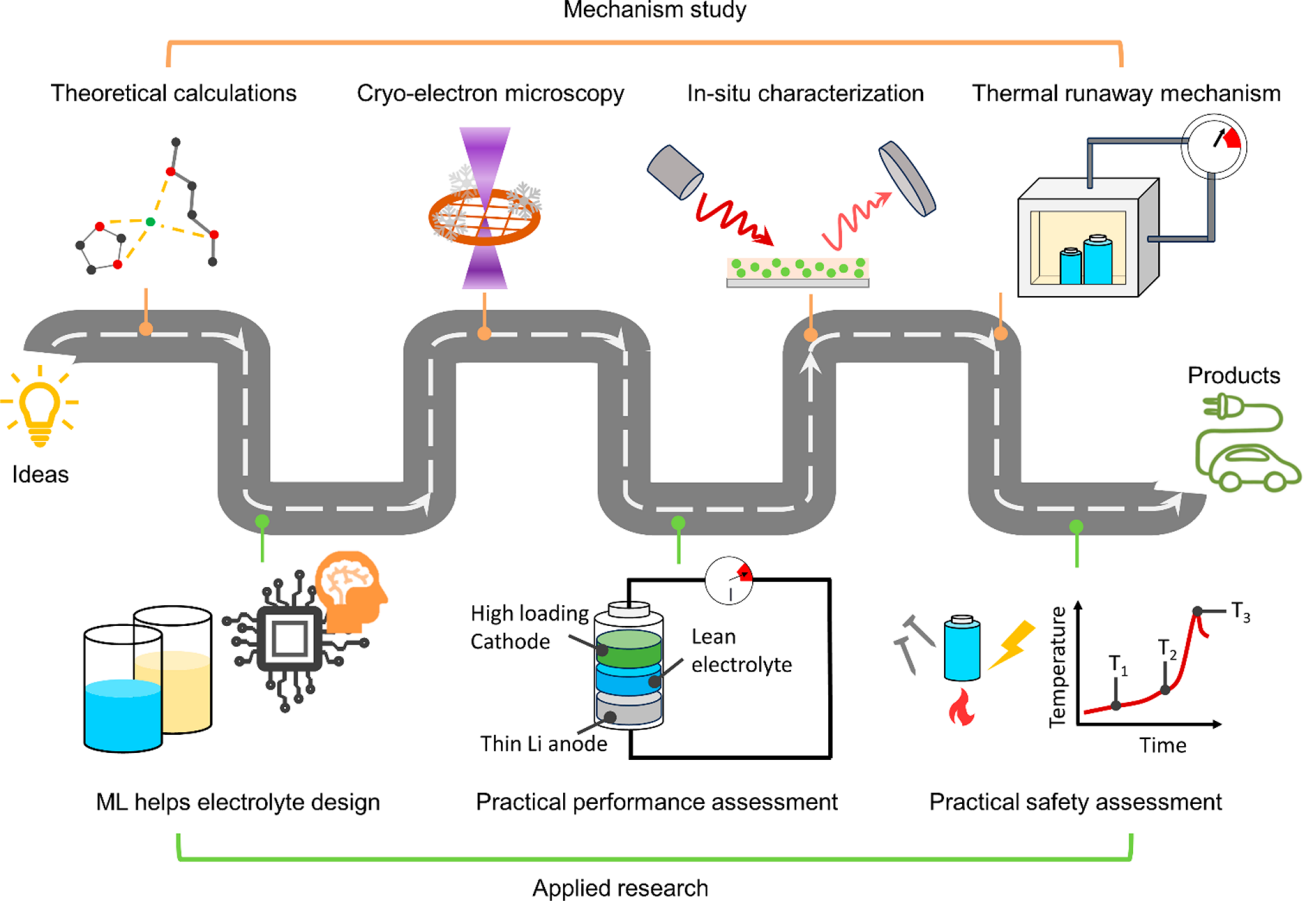
Proposed development roadmap for commercial ether-based HV-LMBs based on mechanism study and applied research.

Compared with traditional LIBs, ether-based HV-LMBs still face many challenges to realize commercial application. These challenges include insufficient cycle life, serious safety concerns, and high production costs. To address the above challenges, we propose a development roadmap (Figure 6): in the future, the ether-based HV-LMBs can be developed simultaneously from both mechanism study and applied research. The proposed development roadmap is as follows:

### Mechanism Study

(1) Investigate the failure mechanisms of CEI and SEI in ether-based HV-LMBs using advanced characterization techniques. Electrolytes have an important effect on the components and structure of CEI and SEI, which is closely related to the performance of ether-based HV-LMBs. Therefore, studying the evolution process of the CEI and SEI during cycling helps understand the degradation mechanisms of battery, thus facilitating the targeted optimization of electrolytes. Theoretical calculations, such as molecular dynamics simulations and density functional theory,^106−109^ can be applied to investigate the oxidative stability of various components of electrolytes in thermodynamics, along with the influence of electrolyte components on solvation structures of electrolytes. Meanwhile, various advanced characterization techniques developed in recent years, such as cryo-electron microscopy,^110,111^ electrochemical quartz crystal microbalance,^112,113^ the time-of-flight secondary ion mass spectroscopy (ToF-SIMS),^49,114^ X-ray photoelectron spectroscopy (XPS),^115,116^ etc., should be applied to conduct an accuracy analysis of the structures of CEI and SEI. This enables the establishment of a relationship between the composition of CEI and SEI and their stability. Additionally, in situ characterization techniques, such as in situ Raman spectroscopy,^117,118^ are important tools for exploring the dynamic changes in the interface structure and composition during cycles.
(2) Analyze the mechanism of TR in ether-based HV-LMBs. The TR in batteries represents a complex process involving various reactions. Taking conventional LIBs as an example, during the TR in batteries, it experiences the decomposition of SEI and lithium salts, exothermic reactions between electrolytes and electrodes, and the electrodes decomposition in sequence, until the battery temperature rises sharply.^39,96^ In contrast to conventional LIBs, ether-based HV-LMBs employ more active Li metal anode instead of graphite and possess a higher upper cutoff voltage, resulting in different chain reactions and more severe exothermic reactions between the electrolytes and electrodes in the LMBs during the TR. Therefore, understanding the mechanism of TR in ether-based HV-LMBs is crucial for developing targeted strategies. Based on this consideration, thermal stability of the designed electrolytes with electrodes should be investigated using differential scanning calorimetry. By combining accelerating rate calorimetry and online electrochemical mass spectroscopy, analyze the chain reactions and generated byproducts in the ether-based HV-LMBs during TR.

### Applied Research

(1) Utilize machine learning (ML) to help design ideal electrolytes for ether-based HV-LMBs. ML makes it possible to accelerate the progress of ether-based electrolytes for HV-LMBs.^119−121^ The performance of batteries is greatly affected by the structure of ether molecules and lithium salts, as well as the concentration of each component of electrolytes. Based on the abundant research results on ether-based electrolytes for HV-LMBs, it is feasible to establish a database related to ether solvents and lithium salts and continually refine it. Relevant parameters influencing the battery performance should be identified and extracted from the database. Then the selected data and characteristic parameters can be used to train the ML models, aiming to guide the design of ideal electrolytes.
(2) Establish standardized criteria for evaluating ether-based HV-LMBs performance. The ether-based HV-LMBs have drawn significant attention because of their high energy densities. However, the performance evaluation standards for the ether-based HV-LMBs need continuous improvement because of the early stage of development in this field. Currently, there is a significant difference between the test parameters in lab and those used in commercial applications in terms of the capacity of Li anode, the negative to positive capacity ratio, and the ratio of electrolyte to capacity. The lenient testing condition may lead to overly optimistic results in this field. Therefore, testing conditions should strive to be consistent with those used in commercial LIBs in the future. Furthermore, there should be a standardized evaluation of the cycling performance of batteries: when the capacity retention of the battery falls below 80%, the battery should be considered as a failure.^122^ The cycle life of the battery should be no less than 500 cycles (according to the frequency of charging the electric vehicle once a week, it can guarantee the service life of the electric vehicle for 10 years).
(3) Improve the safety assessment standards for ether-based HV-LMBs. Battery safety is crucial for the commercialization of ether-based HV-LMBs. In addition to the commonly used self-extinguishing time of electrolyte, the heat generated by electrolytes, electrodes, and the mixture of electrolytes and electrodes is also important parameter for evaluating battery safety.^39,96^ Meanwhile, the self-heating onset temperature (T1), the onset temperature of TR (T2), and the maximum temperature (T3) during TR under various abuse conditions are also significant factors affecting the battery safety. Thus, the above-mentioned parameters need to be comprehensive analyzed in the future research on the safety of ether-based HV-LMBs.

In conclusion, the research on ether-based HV-LMBs is currently in its early stage. Although strategies such as lithium salt optimization, solvent engineering, and functional additives effectively enhance the performance of HV-LMBs, this battery system still suffers from short cycle life, poor safety, and high cost. In the future, it is essential to simultaneously advance mechanistic understanding and practical applications, such as investigating the failure mechanisms of CEI and SEI and the mechanism of TR in ether-based HV-LMBs, establishing standardized protocols for evaluating ether-based HV-LMBs performance, and improving the safety assessment standards. These efforts will lead to synergistic improvement of the electrochemical performance and safety of ether-based HV-LMBs.

## Vocabulary

Li dendrites: Dendritic or mossy protruding structures formed by uneven deposition of Li ions on the surface of Li anode
Energy density: Electrical energy stored per unit volume or mass of active materials or battery
Cathode–electrolyte interphase: The solid interfacial film formed due to the electrolyte decomposition at the cathode interface
Rate performance: Electrochemical performance of batteries at various discharge or charge current densities
Thermal runaway: Uncontrollable and sharp temperature increase of batteries under abusive conditions
